# A Portable Electrochemical Dopamine Detector Using a Fish Scale-Derived Graphitized Carbon-Modified Screen-Printed Carbon Electrode

**DOI:** 10.3390/molecules29030744

**Published:** 2024-02-05

**Authors:** Feng Yang, Xiao Han, Yijing Ai, Bo Shao, Weipin Ding, Kai Tang, Wei Sun

**Affiliations:** 1 Hainan Engineering Research Center of Tropical Ocean Advanced Optoelectronic Functional Materials, Key Laboratory of Laser Technology and Optoelectronic Functional Materials of Hainan Province, Key Laboratory of Functional Materials and Photoelectrochemistry of Haikou, College of Chemistry and Chemical Engineering, Hainan Normal University, Haikou 571158, China; likeyangff@126.com (F.Y.); hanxiao0659@163.com (X.H.); aiyijing1995@163.com (Y.A.); m17264392385@163.com (B.S.); 2 Haikou Marine Geological Survey Center, China Geological Survey, Haikou 571127, China; gzsdingwp@126.com

**Keywords:** electrochemical sensor, screen-printed carbon electrode, graphitized carbon, dopamine

## Abstract

In this paper, a highly conductive alkali-activated graphitized carbon (a-GC) was prepared using tilapia fish scales as precursors through enzymolysis, activation and pyrolytic carbonization methods. The prepared a-GC was modified on the surface of a screen-printed carbon electrode to construct a flexible portable electrochemical sensing platform, which was applied to the differential pulse voltametric detection of dopamine (DA) using a U-disk electrochemical workstation combined with a smart phone and Bluetooth. The prepared a-GC possesses good electrical conductivity, a large specific surface area and abundant active sites, which are beneficial for the electrooxidation of DA molecules and result in excellent sensitivity and high selectivity for DA analysis. Under the optimal conditions, the oxidation peak current of DA increased gradually, with its concentrations in the range from 1.0 μmol/L to 1000.0 μmol/L, with the detection limit as low as 0.25 μmol/L (3S/N). The proposed sensor was further applied to the determination of DA in human sweat samples, with satisfactory results, which provided an opportunity for developing noninvasive early diagnosis and nursing equipment.

## 1. Introduction

Dopamine (DA) is an important neurotransmitter present in the central nervous system of mammals and can affect behavior, emotion and health [1]. The changes in the DA concentration level in the human body will cause a series of diseases, such as Parkinson’s disease, mental abnormality, senile dementia, and emotional disorders [2]. Therefore, the sensitive and accurate measurement of DA is crucial for the prevention and treatment of these diseases. DA can also be found in human sweat, which exhibits positive correlation with its content in blood [3]. Thus, the detection of DA in sweat sample can lead to a non-invasive analysis with real-time monitoring [4,5], which is convenient for disease diagnostics and health control. At present, different technologies, including colorimetric method [6], fluorescence method [7], and surface-enhanced Raman spectroscopy [8], have been used for DA analysis. However, these techniques are often time-consuming and involve expensive instruments, which limits their real-life applications. A focus of the current research is on developing a cost-effective method for the quantitative determination of DA with simple operation and on-site capability. 

Electrochemical sensors have exhibited many advantages, such as high sensitivity, good selectivity and fast response [9]. However, the practical applications of traditional solid electrodes have been limited due to the disadvantages, such as the larger size (diameter of 1–4 mm), complex operation and low repeatability (<20% relative standard deviation) [10]. The combination of a portable electrochemical sensor and a smart phone has been designed with various modifiersand practical applications. For example, Shao et al. [11] designed a portable electrochemical sensing platform with a screen-printed carbon electrode (SPCE) and a Bluetooth electrochemical workstation, which was suitable for the analysis of the indole-3-acetic acid concentration in various tissues of mung bean sprouts. Parrilla et al. [12] modified an SPCE with 1, 2-naphthoquinone-4 sulfonate to construct a sensor for the detection of amphetamine. Chen et al. [13] developed a wireless portable electrochemical sensor based on an SPCE to analyze the indole-3-acetic acid content in different parts of pea seedlings. Zhou et al. [14] developed a high-performance electrochemical sensor based on a reduced graphene oxide and carboxyl-functionalized multi-walled carbon nanotube hybrid-modified SPCE for simultaneous detection of cadmium ion and lead ion. Yamuna et al. [15] prepared a pyrochlore-type lanthanum tin oxide nanoparticle-modified SPCE for carbaryl detection with the limit of detection as 0.4 nmol/L. Nagles et al. [16] developed an ionic liquid-treated chitosan-single-walled carbon nanotube-modified SPCE for simultaneous detection of DA and uric acid (UA). The construction and application of these portable electrochemical sensors will enable the development of point-of-care devices for practical electrochemical applications. 

So far, various nanomaterials, such as metal nanoparticles, metal oxides, carbon nanomaterials and conductive polymers [17,18,19,20], have been used for the oxidation of DA. Among them, carbon nanomaterials have attracted increasing attention due to the good conductivity (>500 S/m) [21] and adjustable physical/chemical properties. Biomass-derived carbon, a kind of renewable carbon materials obtained from animal and plant wastes, is widely used in electrochemical sensing applications because of the high specific surface area, rich porous structure, superior physical and chemical stability [22]. For example, Zhang et al. [23] constructed an electrochemical sensing platform based on a composite combination of Kiwi skin-derived carbon and zinc chloride nanoparticles, which was used to simultaneously detect ascorbic acid (AA), DA and UA, with the corresponding detection limits (S/N = 3) of 0.02 μM, 0.16 μM, and 0.11 μM. Mahmood et al. [24] converted biomass-based film composed of kraft lignin and cellulose nanofibers into porous graphene and used it for the voltametric detection of DA, which demonstrated that biomass-based material had the potential to be transformed into carbon materials with good electrochemical performance. Padmapriya et al. [25] prepared a phosphorus and nitrogen dual-doped carbon quantum dot-modified electrode with the banana flower bract extract as a precursor and used it for DA detection in the ultralow detection limit (picomolar). Sha et al. [26] synthesized a shaddock peel-derived carbon nanoball aggregation network-based aerogel, which had a large surface area (446.39 m^2^/g) and hierarchical meso-microporous structure, and may provide a favorable path for electrolyte penetration and transportation. The above reports indicate the great promise of biomass-derived carbon for the electroanalysis, and it remains to be studied to further improve the electroanalysis performance of sensors. 

High-temperature pyrolysis, catalytic pyrolysis and alkali activation are commonly used methods to adjust the morphological structure and graphitization degree of biomass carbon [27,28,29,30,31]. The graphitization degree of biomass carbon affects the defect site density and conductivity [32]; thus, it is a good strategy to improve the electrochemical performance by adjusting the graphitization degree of biomass carbon. Cheng et al. [33] prepared a highly graphitized *N*-doped porous carbon material derived from biomass with ferric ammonium citrate as an active agent. He et al. [34] prepared microporous carbon with a large specific surface area (2208 m^2^/g) and high conductivity (2.38 S/cm) by controlling the activation temperature of biomass pyrolysis with potassium ferrate as a catalyst and activation agent. Zhao et al. [35] prepared graphene-like nitrogen-doped carbon using chitosan as a precursor and ferric chloride as a soft template. Therefore, biomass-derived porous carbons with a high graphitization degree, large specific surface area and high conductivity are expected to achieve excellent sensing performance.

**Scheme 1:**
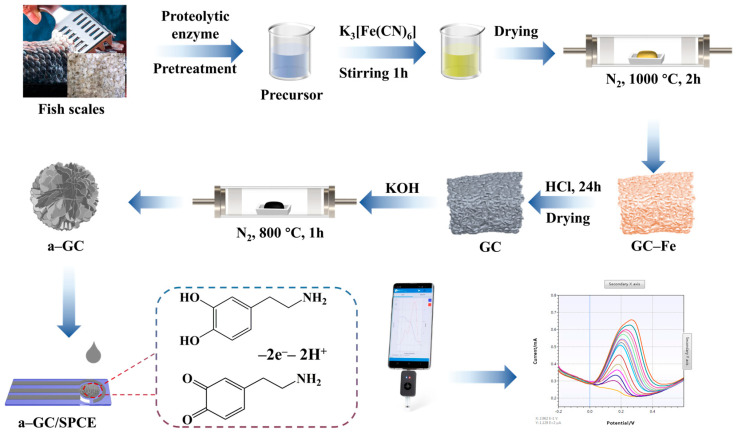
Synthesis of a-GC and electrochemical detection process for DA using a U-disk electrochemical workstation.

In this paper, alkali-activated graphitized carbon (a-GC) was prepared using fish scales as precursors via enzymolysis, activation and the two-step pyrolytic carbonization method, with K_3_[Fe(CN)_6_] as the catalyst. By employing an a-GC-modified SPCE as the base electrode, rapid and sensitive detection of DA was realized on the U-disk electrochemical workstation combined with a smart phone through Bluetooth. The preparation process for the a-GC and the application in portable electrochemical sensor are illustrated in Scheme 1, with the detailed procedures described in Section 3. The fish scales used in the experiment are tilapia scale wastes that are abundant in Hainan Province of China owing to its well-developed fishery industry, where low-value biomass waste is converted into high-value products in large-scale preparation through pyrolysis technology. As demonstrated below, graphitized carbon obtained via this process shows higher electrical conductivity, which is very promising both in the sensing platform as a modified electrode material and in other energy conversion and storage fields. The results in the paper provide some insights for the recycling of fish scales and the development of novel functional carbon materials from fishery waste.

## 2. Results and Discussion

### 2.1. Characterizations of a-GC

**Figure 1:**
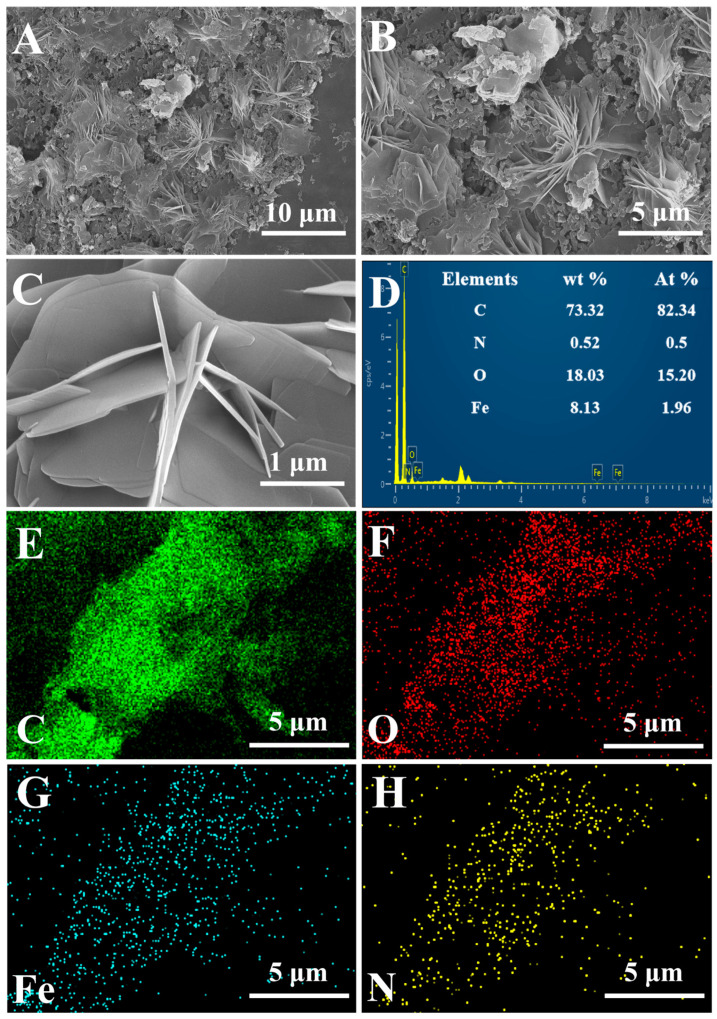
(**A**–**C**) Scanning electron microscopy images of a-GC at 10,000×, 20,000× and 100,000× magnifications, (**D**) energy dispersive X-ray spectroscopy spectrogram of a-GC, elemental mapping of (**E**) C, (**F**) O, (**G**) Fe and (**H**) N of a-GC.

The morphological features of a-GC produced via two-step carbonization were characterized by means of a field emission scanning electron microscope (FESEM) with the corresponding results obtained at 10,000×, 20,000× and 100,000× magnifications shown in Figure 1A–C. The gradually enlarged SEM image indicates the prepared a-GC exhibited a flower-like structure with a diameter of ~5 μm, which was formed by the staggered accumulation lamellas with a thickness of ~36 nm. The flower-like structure of a-GC as an electrode material may greatly enhance the effective electrode area and facilitate the diffusion of analytes in the electrochemical processes. The scanning electron microscopic–energy dispersive X-ray spectroscopic results in Figure 1D show the presence of C, N, O and Fe in a-GC. Notably, the Fe content of 8.13 wt% might have originated from the incomplete removal of iron from the GC-Fe, which might enhance the electrocatalytic activity and affinity of a-GC through the chelation of Fe and DA [36]. Elemental mapping micrographs of C (Figure 1E), O (Figure 1F), Fe (Figure 1G) and N (Figure 1H) indicate that all the elements were uniformly distributed on the surface of the a-GC.

**Figure 2:**
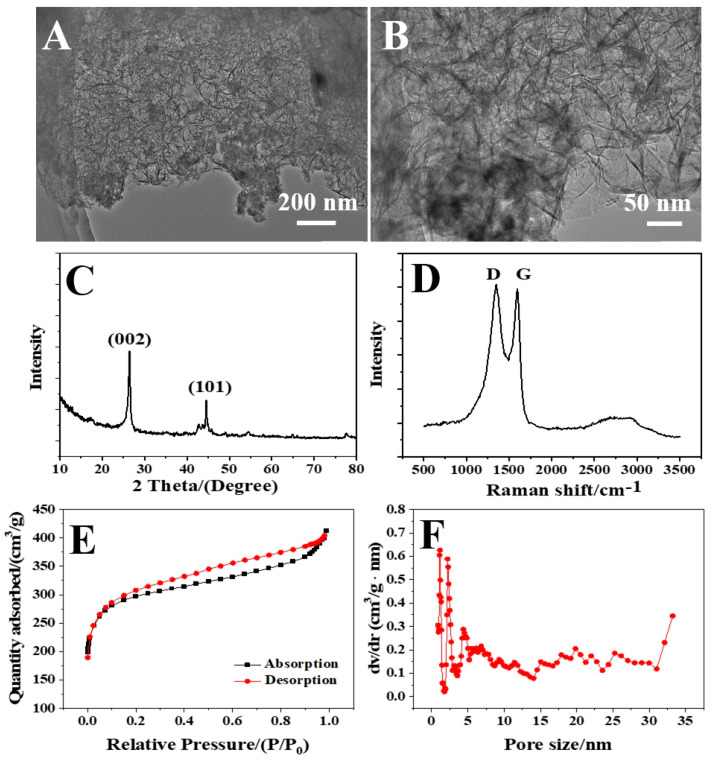
(**A**,**B**) Transmission electron microscopy images at 30,000× and 100,000× magnification, (**C**) XRD pattern, (**D**) Raman spectrum, (**E**) the N_2_ adsorption and desorption isotherm and (**F**) the pore size distribution curve of a-GC.

The morphological structures of a-GC at different magnifications were investigated using transmission electron microscopy (TEM) at 30,000× and 100,000× magnifications, with the results shown in Figure 2A,B. The a-GC displayed an obvious layered structure, which could increase the specific surface area and then provide more attachment points for further interaction with DA. The X-ray diffractometer (XRD) pattern of the a-GC (Figure 2C) showed a sharp peak at 26.42° and a broad diffraction peak at 44.39°, which corresponded to the (002) lattice plane and the (101) phase of graphitized carbon, confirming the existence of graphitized carbon obtained via a solid-state pyrolysis process [25]. 

The graphitic structure could be further verified via the Raman spectroscopic result (Figure 2D), in which the D and G peaks were located at 1350 cm^−1^ and 1580 cm^−1^, with the intensity ratio (I_D_/I_G_) as 1.02. Generally, it is believed that the D band originates from the defects of graphite and G band is thought to represent the degree of graphitization [37]. The I_D_/I_G_ value indicated that the GC-a had a graphitization degree with some defects that may be ascribed to the N-doping. Therefore, fish scale-based biomass carbon with a high graphitization degree has been successfully synthesized using the two-step carbonization and KOH activation process.

The pore size distribution and surface area of the a-GC were further investigated via Brunauer–Emmett–Teller (BET) and N_2_ adsorption–desorption measurements. As shown in Figure 2E, a-GC exhibited a type II isotherm and hysteresis loop in the relative equilibrium pressure (P/P_0_) range of 0–1.0, indicating a mainly microporous structure. In addition, the BET specific surface area of the a-GC was 1093.5 m^2^/g, with the major peak of pore size distributed in 1.1 nm and 2.2 nm (Figure 2F), which indicated that the a-GC possessed abundant micropores (<2.0 nm) and mesopores (from 2.0 to 50 nm). The presence of microporous will facilitate electron transfer and DA adsorption. Moreover, the mesopores offer a larger active area and good corridor structure for the adsorption of DA molecules, which may be beneficial to the diffusion of DA molecules on the surface of the modified electrode.

### 2.2. Electrochemical Properties of a-GC/SPCE

**Figure 3:**
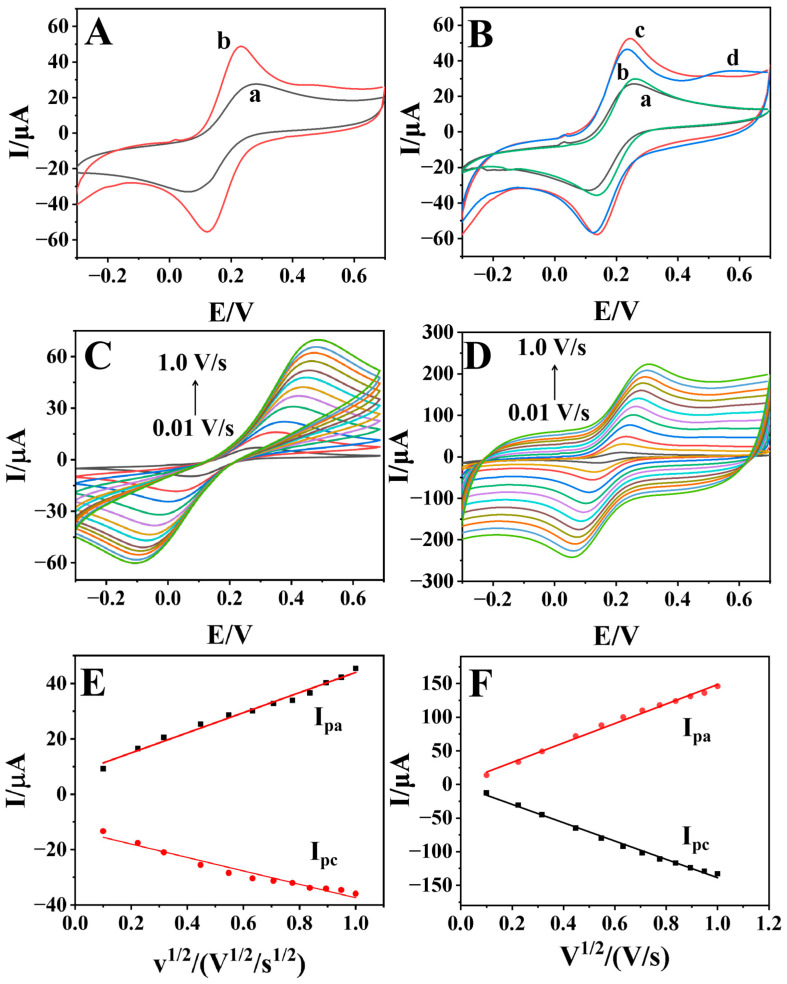
Cyclic voltammograms of (**A**) different modified electrodes (a: SPCE, b: a-GC/SPCE) and (**B**) different concentrations of a-GC modified on SPCE (a–d: 0.5, 1.0, 1.5, 2.0 mg/mL) in 1.0 mmol/L [Fe(CN)_6_]^3−^ and 0.5 mol/L KCl solution at a scan rate of 0.1 V/s; cyclic voltammograms of (**C**) SPCE and (**D**) a-GC/SPCE at different scan rates (0.01, 0.05, 0.1, 0.2, 0.3, 0.4, 0.5, 0.6, 0.7, 0.8, 0.9, 1.0 V/s) in 1.0 mmol/L [Fe(CN)_6_]^3−^ and 0.5 mol/L KCl solution, and the corresponding linear relationship between I_p_ and υ^1/2^ of (**E**) SPCE and (**F**) a-GC/SPCE.

The electrochemical performances of working electrodes are often checked using the redox responses of [Fe(CN)_6_]^3−^, which is a commonly used probe to evaluate the electrochemical properties of the modifier on the surface of a working electrode. Therefore, the electrochemical behaviors of 1.0 mmol/L [Fe(CN)_6_]^3−^ at an SPCE and an a-GC/SPCE in a 0.5 mol/L KCl-supporting electrolyte were studied via cyclic voltammetry (CV) at a scan rate of 0.1 V/s. As shown in curve a in Figure 3A, an oxidation peak of 29.46 μA (I_pa_) at 0.25 V and a reduction peak of 27.68 μA (I_pc_) at 0.11 V were observed. Meanwhile, on an a-GC/SPCE (curve b), the I_pa_ and I_pc_ increased to 44.65 mA and 48.64 mA, which could be ascribed to the active surface area and accelerated electron transfer rate of the a-GC on the electrode surface. 

In order to optimize the quantity of a-GC immobilized on the electrode surface, CV curves of SPCE modified with an increasing concentration of a-GC suspension from 0.5, 1.0, 1.5 and 2.0 mg/mL were recorded in 1.0 mmol/L [Fe(CN)_6_]^3−^. As shown in Figure 3B, the redox peak current increased from 27.04 μA to 52.54 μA as the concentration of the a-GC suspension increased from 0.5 mg/L to 1.5 mg/L. However, when the concentration was further increased to 2.0 mg/mL, the redox peak current was observed to decrease to 46.47 μA, which may be due to the increase in the thickness of the a-GC film on the electrode surface. Also, the background peak increased on the a-GC/SPCE at a higher a-GC concentration, which could be ascribed to the increase in the thickness of the a-GC film with large double-layer charged current. As a result, 25 µL 1.5 mg/mL a-GC suspension was selected as the optimal quantity of modifier for the further electrochemical investigations.

To explore the electrochemical effective area of SPCE and a-GC/SPCE, CV curves were recorded in 1.0 mmol/L [Fe(CN)_6_]^3−^ with a scan rate range of 0.01–1.0 V/s. As depicted in Figure 3C,D, the redox peak currents increased gradually with the increase in the scan rate. A good linear relationship between the redox peak current (I_p_) and the square root of the scan rate (υ^1/2^) could be achieved with the coefficient as 0.99, which indicated a diffusion-controlled electrode reaction process (Figure 3E,F). The electrochemical effective areas of the SPCE and a-GC/SPCE were further estimated according to Randles–Sevcik formula [38], with the results of 0.053 cm^2^ and 0.208 cm^2^, respectively. The effective area of a-GC/SPCE was 3.92 times higher that of SPCE, which might be related to the presence of the porous structure of a-GC with a large surface area on the electrode surface.

### 2.3. Electrochemical Behaviors of DA on a-GC/SPCE

**Figure 4:**
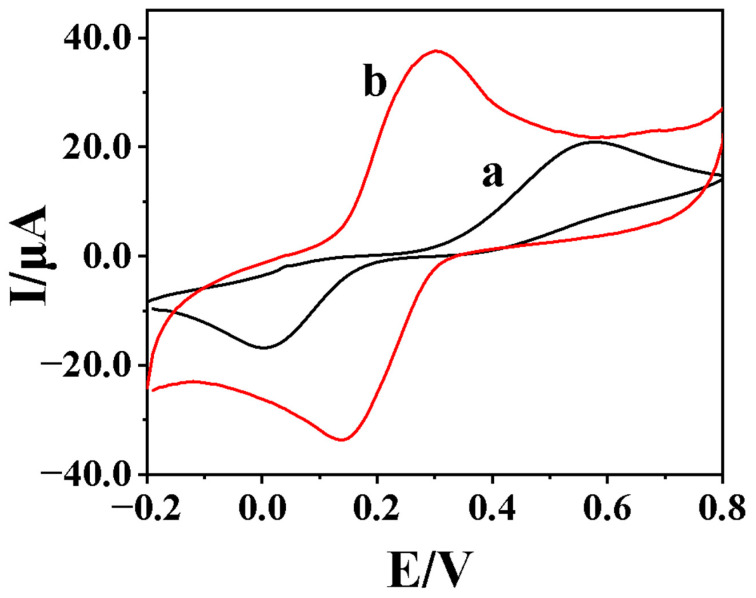
Cyclic voltammograms of 0.5 mmol/L DA on (a) SPCE and (b) a-GC/SPCE in 0.1 mol/L PBS (pH 6.0); scan rate of 0.1 V/s.

DA is an important neurotransmitter with a hydroxyl functional group in the molecular structure, which can be electro-oxidized on the electrode surface. The electrochemical behaviors of DA on different modified electrodes were investigated in 0.1 mol/L phosphate buffered saline (PBS, pH 6.0) containing 0.5 mmol/L DA, with the results shown in Figure 4. In the voltammogram obtained at a bare SPCE (curve a), an oxidation peak of 20.89 μA at 0.57 V (E_pa_) and a reduction peak of 16.76 μA at 0.00 V (E_pc_) were observed, which corresponded to a peak separation (ΔE_p_) of 0.57 V. Similarly, the CV obtained at an a-GC/SPCE (curve b) shows a I_pa_ of 37.22 μA at 0.28 V and a I_pc_ of 33.37 μA at 0.14 V, giving an ΔE_p_ of 0.14 V. The decrease in the ΔE_p_ value (from 0.57 V to 0.14 V) proved that a-GC could provide excellent conductivity and high surface areas for the redox of DA. And these results might be because that the a-GC with layered structures can effectively reduce the diffusion length of ions from the electrolyte and provide additional active area. In addition, a significant shift in the oxidation peak to the lower potential with improved peak currents was observed on a-GC/SPCE, which could also be ascribed to the electrocatalytic activity and fast electron transport efficiency of a-GC. 

### 2.4. Electrochemical Investigations of DA on a-GC/SPCE 

**Figure 5:**
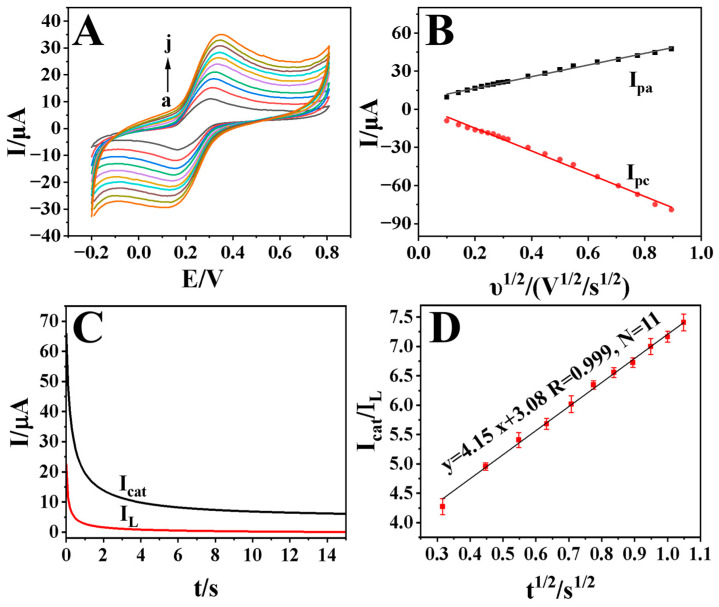
(**A**) Cyclic voltammograms of a-GC/SPCE at different scan rates (a–j: 0.01, 0.02, 0.03, 0.04, 0.05, 0.06, 0.07, 0.08, 0.09, 0.1, 0.15, 0.2, 0.25, 0.3, 0.35, 0.4, 0.45, 0.5 V/s) in 0.1 mmol/L DA and PBS (pH 6.0) and (**B**) the corresponding relationship of I_p_ versus υ^1/2^, (**C**) chronoamperometric response of a-GC/SPCE in 0.1 mol/L PBS (pH 6.0) in the absence (I_L_) and presence (I_cat_) of 0.1 mmol/L DA, (**D**) the corresponding relationship of I_cat_/I_L_ versus t^1/2^.

The influence of the scan rate on the cyclic voltametric response of 0.1 mmol/L DA was investigated using GC-a/SPCE in 0.1 mol/L PBS (pH 6.0) and the results are shown in Figure 5A. It can be seen that the increase in the scan rate from 0.01 V/s to 0.5 V/s led to the increase in the redox peak current. As depicted in Figure 5B, the redox peak current is directly proportional to the square root of the scan rate (υ^1/2^), with the linear regression equations set as I_pa_ (μA) = 45.4 υ^1/2^ (V/s) −7.49 (R = 0.995, N = 10) and I_pc_ (μA) = −87.2 υ^1/2^ (V/s) + 2.32 (R = 0.994, N = 10), which indicated a diffusion-control reaction process of DA on a-GC/SPCE. The relation between the I_pa_ (μA) and the square root of scan rate (υ^1/2^) is given by the Randles–Sevcik equation: I_pa_ = (2.69 × 10^5^) n^3/2^ AC_0_D_0_^1/2^ υ^1/2^, where n is the number of electrons exchanged in oxidation at T = 298 K, A is the effective electrode area (0.208 cm^2^), C_0_ is the DA concentration (1.0 × 10^−4^ mol/L), D_0_ is the diffusion coefficient of DA (7.30 × 10^−6^ cm^2^/s [39,40]), and υ is the scan rate (V/s). In this way, n is evaluated as 2.08, which implies a two-electron transfer process on the a-GC/SPCE.

To further evaluate the electrocatalytic parameter and electrochemical kinetic of DA on the surface of a-GC/SPCE, the chronoamperometric responses on a-GC/SPCE were investigated in the absence (I_L_) and presence (I_cat_) of 0.1 mmol/L DA solution at the potential of 0.5 V (Figure 5C). The catalytic rate constant (k_cat_) of DA oxidation could be obtained using the following equation [41]: I_cat_/I_L_ = (πk_cat_*C*_0_ t)^1/2^, where I_cat_ and I_L_ are the current on the a-GC/SPCE in the presence and absence of DA, respectively, and t is the elapsed time. From the slope of the I_cat_/I_L_ vs. t^1/2^ plot (Figure 5D), the value of k_cat_ was found to be 5.48 × 10^4^ M^−1^ s^−1^, which was obviously larger than that reported in the literature (8.81 × 10^3^ M^−1^ s^−1^ [42], 2.57 × 10^4^ M^−1^ s^−1^ [43], 6.80 × 10^2^ M^−1^ s^−1^ [44]). The results prove that a-GC has good electrocatalytic activity for electrochemical oxidation of DA. 

**Figure 6:**
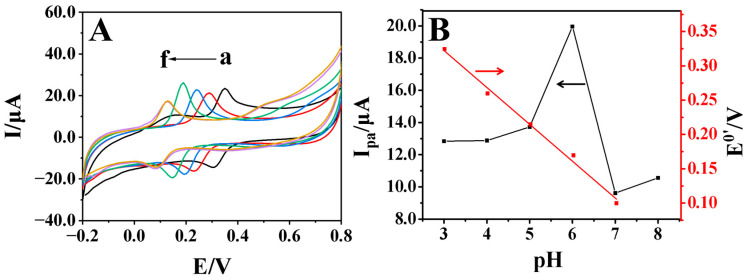
(**A**) Cyclic voltammograms of a-GC/SPCE in 0.1 mmol/L DA with different pH values (a→f: pH 3.0, 4.0, 5.0, 6.0, 7.0, 8.0), (**B**) the plot of I_pa_ (black line) and E^0^’ (red line) of DA versus pH.

The influence of the pH of PBS was studied based on the CV of 0.1 mmol/L DA on a-GC/SPCE in 0.1 mol/L PBS with the pH value varying from 3.0 to 8.0. The results obtained are depicted in Figure 6A. Here, the oxidation peak of DA was observed to shift negatively from 0.35 V to 0.14 V, and the reduction peak to shift from 0.31 V to 0.09 V, with the increase in the pH from 3.0 to 7.0, indicating that protons were involved in the electrode reaction. As shown in Figure 6B, a linear relationship between the formal peak potential (E^0^’; estimated using the average of E_pa_ and E_pc_) and pH was obtained with the linear regression equation set as E^0^’(V) = 0.48 − 0.054 pH (R = 0.993, N = 5). The slope value of 54 mV/pH was close to the theoretical value of 59 mV/pH for an equal number of protons and electrons involved in the electrode reaction. The oxidation peak current increased gradually with the increase in the pH from 3.0 to 6.0, and it reached a maximum value at the pH value of 6.0 and then decreased at pH 7–8, so pH 6.0 was selected for further investigation. The decrease in the oxidation peak current at pH 7–8 was likely because DA could self-polymerize into polydopamine in neutral and alkaline solutions [45,46]. DA can be polymerized in the electrolyte under alkaline conditions, which led to the decrease in the concentration of free DA in the solution, resulting in a decrease in the redox peak current.

**Scheme 2:**
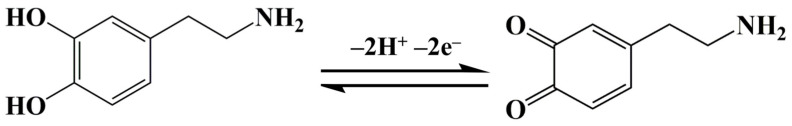
Electrochemical oxidation mechanism of DA on a-GC/SPCE.

As the electrooxidation process is isoelectronic and isoprotonic, DA exhibits a two electrons and two protons process on a-GC/SPCE. The oxidation mechanism is shown in Scheme 2, in which DA loses two electrons and two protons to become dopamine-o-quinone. Then, the reduction reaction occurs according to the reverse process.

### 2.5. Calibration Study for DA Analysis

**Figure 7:**
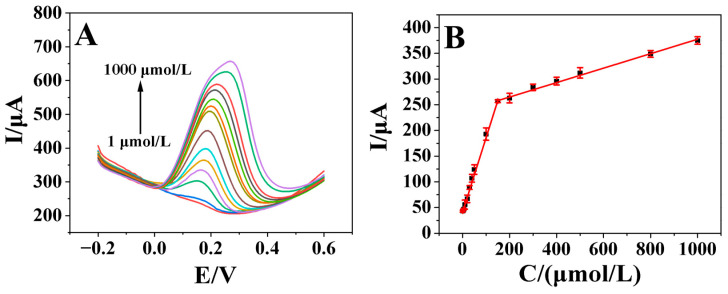
(**A**) Differential pulse voltammograms of a-GC/SPCE in different concentrations of DA and (**B**) the relationships of the oxidation peak currents with the DA concentrations.

The differential pulse voltammetry (DPV) method was used for the quantitative analysis of DA in this study. As shown in Figure 7A, the oxidation peak current increased gradually with the DA concentrations ranging from 1.0–1000.0 μmol/L. Good linear relationships were obtained between the oxidation peak currents and DA concentration (Figure 7B) in the range from 1.0–150.0 μmol/L and 150.0–1000.0 μmol/L, with the linear regression equations set as I_1_(μA) = 1.50 C_DA_ (μmol/L) + 43.2 (R = 0.996, N = 8) and I_2_(μA) = 0.100 C_DA_ (μmol/L) + 241 (R = 0.995, N = 7). The sensitivity (slope) of the calibration plot decreased from 1.5 μA/(μmol/L) to 0.1 μA/(μmol/L) when the DA concentration was higher than 150.0 μmol/L, which might be due to the active sites on the surface of a-GC/SPCE decreasing relative to the total number of DA molecules [47]. Based on a signal-to-noise ratio of 3, the limit of detection (LOD) was estimated to be 0.25 μmol/L using the linear calibration results between 1.0 and 150.0 μmol/L. The analytical parameters of DA using different modified electrodes and detection methods are listed in Table 1, which indicates that a-GC/SPCE has a comparable LOD and wider linear range with others. The wider linear range may be due to the large surface area and more electroactive sites of a-GC, which is conducive to the adsorption and reaction of DA molecules. In addition, a-GC/SPCE exhibits considerable merits, including low-cost raw materials and portable detection on smart phones with a small size U-disk electrochemical workstation, which is more suitable for the point-of-care testing.

### 2.6. Selectivity, Repeatability and Stability

**Figure 8:**
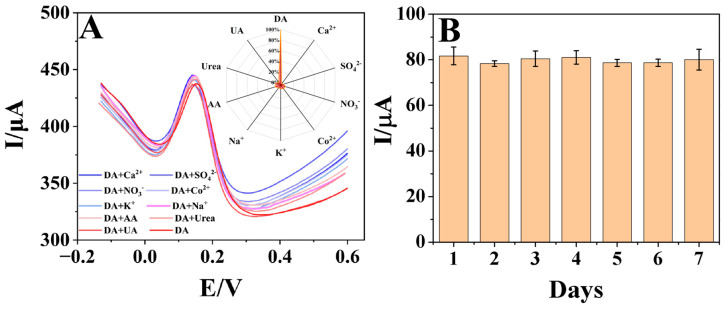
(**A**) Differential pulse voltammograms of a-GC/SPCE in 25.0 μmol/L DA solution containing 50.0 μmol/L interfering substance (inset is the corresponding selectivity), and (**B**) the oxidation current values of a-GC/SPCE in 25.0 μmol/L DA for 7 days.

In order to investigate the selectivity of a-GC/SPCE, differential pulse voltammograms of 25.0 μmol/L DA containing 50.0 μmol/L common interferents (such as Ca^2+^, SO_4_^2−^, NO_3_^−^, K^+^, Na^+^, AA, urea and UA) were recorded. As shown in Figure 8A, there was no significant influences on the oxidation currents of DA in the presence of these interferents (with the signal change of less than 6%), which proved that a-GC/SPCE had good selectivity. And the change in the current value after adding 50.0 μmol/L common interferences were normalized to 1 and plotted in the radar graph of Figure 8A in order to visualize the selectivity of a-GC/SPCE. Multi-sweep cyclic voltammetry was used to record 50 cycles of five a-GC/SPCE. The results showed that the currents of the five electrodes were basically unchanged. After the modified electrode was stored at room temperature for 7 days, the peak current measured in 25.0 μmol/L DA solution remained 97.56% of the initial value with the relative standard deviation (RSD) of the current less than 4.6% (Figure 8B). The above results indicated that the sensor had good selectivity, stability and reproducibility for DA analysis.

### 2.7. Real-Life Sample Analysis

As compared with other body fluids, sweat has advantages such as easy sampling, simplicity of operation and obtainment; thus, human sweat was used as the real-life sample to explore the reliability of a-GC/SPCE. The sweat sample was diluted 10 times with 0.1 mol/L PBS (pH 6.0) and a standard addition calibration method was performed to estimate the DA concentration. After three parallel experiments were conducted to detect the concentration of DA, the results obtained through calibration are listed in Table 2. The recovery was 98.90~102.30% via the standard addition method with a calculated RSD value less than 3.0%. Therefore, a-GC/SPCE has potential application prospects in the actual detection of DA.

## 3. Experimental

### 3.1. Instruments and Reagents

Dopamine (DA > 98%) were obtained from Shanghai Aladdin Reagent Co., Ltd., (Shanghai, China). Tilapia fish scales were acquired from local supermarkets. Potassium ferricyanide (K_3_[Fe(CN)_6_]) were purchased from Guangzhou Chemical Reagent Factory (Guangzhou, China). Sweat samples were collected from volunteers’ faces following vigorous exercise using 1.5 mL centrifuge tubes. These samples were then filtered using a 0.22 μm syringe filter before being stored at 4 °C for further examination. In our work, 0.1 mol/L PBS (pH 6.0) was used as a supporting electrolyte. All the reagents were of analytical grade and all the solutions were prepared with ultrapure water (specific resistance of 18 MΩ·cm) obtained from a Milli-Q Plus system (Millipore, IQ7000, Merck Millipore Co., Ltd., Burlington, MA, USA).

Electrochemical experiments were conducted using a U-disk electrochemical workstation (Sensit Smart U disk, Palmsens Co., Ltd., Houten, The Netherlands), which was connected and controlled via a Huawei smart phone with Bluetooth. The SPCE is integrated with a three-electrode system comprising a carbon ink-printed electrode (5.0 mm diameter) as a working electrode, an Ag/AgCl reference electrode, and a carbon counter electrode, which was provided by Weihai Potential Technology Co., Ltd. (Weihai, China). The morphologies and elemental analysis of a-GC were recorded via SEM (Apreo SEM, Thermo Fisher Scientific, Trondheim, Norway) coupled with EDS (X-Max, Oxford instruments, Abingdon, UK), and the microstructures were characterized using field emission TEM (JEM-2100F, Japan Electronics Co., Ltd., Tokyo, Japan). XRD (X-pert 3, PANalytical B.V., Almelo, The Netherlands) was applied to study the crystal phase structure of a-GC with a Cu Kα radiation at wavelength of 1.5406 Å. The Raman spectrum in the range from 500 to 3500 cm^−1^ was obtained on a LabRAM HR system using a 532 nm laser (Horiba, Palaiseau, France). The N_2_ adsorption and desorption isotherms, surface area and pore size distribution of a-GC were studied on a Physical Adsorption Instrument (ASAP 2020, Micromeritics Co., Phoenix, AZ, USA).

### 3.2. Synthesis of a-GC

The carbon precursor was prepared using an enzymatic hydrolysis method previously reported by our group [27]. Briefly, tilapia fish scales were sequentially soaked in a concentration of HCl and 10 wt% NaOH to remove inorganic salts and hydroxyapatite. Then, 12.5 g of the treated product was added to a 250 mL aqueous solution containing 3.0 g alkaline protease, and then stirred at 75 °C for 12 h. The product was filtered using a 0.45 μm and a 0.2 μm filter membrane, and heated at 75 °C for 4 h with magnetic stirring to obtain a homogeneous concentrated carbon precursor solution.

Next, 0.33 g K_3_[Fe(CN)_6_] was dissolved homogeneously in 30 mL precursor and further heated until the solvent was completely evaporated. The dried solid was transferred to a tube furnace and carbonized in N_2_ at 1000 °C for 2 h, with a heating rate of 5 °C /min. The obtained sample was washed with ultrapure water several times and dried in a vacuum at 100 °C for 12 h to obtain GC-Fe. The GC-Fe was soaked in 36 wt% HCl for 24 h at room temperature to remove iron, then the solid was washed with ultrapure water several times to a neutral pH, and dried at 80 °C overnight to obtain the black solid named GC.

The alkaline activation treatment was performed with the following procedure. GC and KOH were mixed evenly at a mass ratio of 1:4, and the mixture was held in an 800 °C tube furnace with an N_2_ atmosphere for 1 h. Finally, the product was washed with ultrapure water to neutrality, and then dried at 60 °C overnight to obtain alkali-activated graphite carbon, which was denoted as a-GC.

### 3.3. Electrochemical Investigations

In our work, 1.5 mg/mL a-GC suspension was prepared in ultrapure water. Then, 25 μL of the above suspension was applied to the SPCE surface and dried at room temperature to obtain a-GC/SPCE. The electrochemical behaviors of DA on different modified electrodes were studied by means of CV, DPV and chronoamperometry using a U-disk workstation connected with a smart phone with Bluetooth and Android system. The CV parameters were set in the potential range of −0.2~0.8 V, with the scan speed set as 0.1 V/s. DPV was carried out with the following parameters: potential range from −0.2 to 0.6 V, pulse width of 50 ms, pulse increment of 10 mV, pulse amplitude of 50 mV. Chronoamperometry was measured at an initial potential of 0.2 V, interval time of 0.1 s and run time of 15 s.

## 4. Conclusions

In this paper, fish scales were handled with enzymolysis, carbonization and activation to synthesize the a-GC. The electrochemical characterizations showed that the a-GC derived from fish scales can act as an excellent modifier for electrochemical sensors. In addition, activation and graphitization have effectively improved the porosity and graphitization degree of biomass carbon, which was demonstrated to enhance the electrochemical performances of the modified electrode. The a-GC-modified SPCE was further used to construct a portable electrochemical sensor for DA detection by combining it with the U-disk electrochemical workstation and a smart phone with two linear ranges of 1.0–150.0 μmol/L and 150.0–1000.0 μmol/L. Based on a signal-to-noise ratio of 3, a limit of detection of 0.25 μmol/L was estimated. The proposed method was successfully applied to the quantitative detection of human sweat samples, with satisfactory results, which provided an opportunity for developing noninvasive diagnosis and nursing equipment of DA.

## Figures and Tables

**Table 1:** Comparison of the analytical performances of different electrochemical DA sensors.

Electrodes	Methods	Linear Range (μmol/L)	LOD (μmol/L)	References
Au@Pt/GO/GCE	DPV	1.0–100.0	5.0	[48]
CA/GCE	CV	5–100	0.86	[49]
LS-rGO/GCE	DPV	0.12–100	0.035	[50]
Pd-NPs-rGO/GCE	LSV	1–150	0.233	[51]
Graphene/GCE	DPV	4–100	2.64	[52]
Nafion/CCINPs-CS/GCE	DPV	2.0–60	1.0	[53]
N-rGO/GCE	DPV	1–60	0.1	[54]
rGS-GNP/Au	DPV	0.1–100	0.098	[55]
f-MWCNT/AgNP/GCE	DPV	1–8	0.2778	[56]
GQDs-MWCNTs/GCE	DPV	0.05–100.0	0.00087	[57]
PtAu/GCE	DPV	24–384	24	[58]
a-GC/SPCE	DPV	1.0–150.0 150.0–1000.0	0.25	This work

Note: GO: graphene oxide; GCE: glassy carbon electrode; CA: catechol; LS: lignosulfonate; rGO: reduced graphene oxide; Pd-NPs: platinum nanoparticles; CCINPs-CS: carbon-coated iron nanoparticles and chitosan composite; N-rGO: nitrogen-doped reduced graphene oxide; rGS: reduced graphene oxide sheet; GNP: gold nanoparticle; MWCNT: multi-walled carbon nanotube; GQDs: graphene quantum dots.

**Table 2:** Detection results of DA in human sweat samples.

Samples	Added (μmol/L)	Found (μmol/L)	Recovery (%)	RSD (%)
Human sweat	10.00	10.23	102.30	2.48
	20.00	19.78	98.90	1.43
	30.00	29.76	99.20	2.29

## Data Availability

Data are contained within the article.
